# Emerging Roles for Neuropilin-2 in Cardiovascular Disease

**DOI:** 10.3390/ijms21145154

**Published:** 2020-07-21

**Authors:** Jennifer L. Harman, Jacob Sayers, Chey Chapman, Caroline Pellet-Many

**Affiliations:** 1 Department of Comparative Biomedical Sciences, Royal Veterinary College, Royal College Street, London NW1 0TU, UK; cnchapman@rvc.ac.uk; 2 University College London, Division of Medicine, Rayne Building, University Street, London WC1E 6JF, UK; jacob.sayers@ucl.ac.uk

**Keywords:** neuropilin, atherosclerosis, inflammation, endothelial cell, macrophages, vascular smooth muscle, cell surface receptor, drug target

## Abstract

Cardiovascular disease, the leading cause of death worldwide, is predominantly associated with atherosclerosis. Atherosclerosis is a chronic inflammatory disease characterised by the narrowing of large to medium-sized arteries due to a build-up of plaque. Atherosclerotic plaque is comprised of lipids, extracellular matrix, and several cell types, including endothelial, immune, and vascular smooth muscle cells. Such narrowing of the blood vessels can itself restrict blood flow to vital organs but most severe clinical complications, including heart attacks and strokes, occur when lesions rupture, triggering the blood to clot and obstructing blood flow further down the vascular tree. To circumvent such obstructions, percutaneous coronary intervention or bypass grafts are often required; however, re-occlusion of the treated artery frequently occurs. Neuropilins (NRPs), a multifunctional family of cell surface co-receptors, are expressed by endothelial, immune, and vascular smooth muscle cells and are regulators of numerous signalling pathways within the vasculature. Here, we review recent studies implicating NRP2 in the development of occlusive vascular diseases and discuss how NRP2 could be targeted for therapeutic intervention.

## 1. Introduction

**Figure 1:**
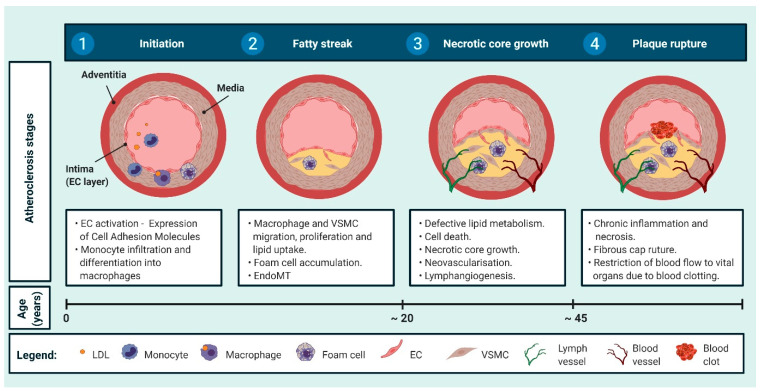
Four main stages of atherogenesis. (**1**) In response to endothelial cell (EC) activation, circulating monocytes adhere to the vascular wall and infiltrate the intima. In the intima, monocytes mature to macrophages and uptake lipids to become foam cells. (**2**) Vascular smooth muscle cells (VSMCs) migrate, proliferate, and secrete extracellular matrix (ECM) proteins. VSMCs and macrophages uptake lipid to form foam cells leading to fatty streak formation. ECs undergo EndoMT. These two initial stages can occur by puberty. (**3**) Extracellular lipids released from dead and dying VSMCs and macrophages accumulate at the centre of the plaque to form the necrotic core. VSMCs on the luminal side of the vessel wall secrete ECM, forming the fibrous cap, and provide stability to the lesion. Neovascularisation and lymphangiogenesis may also occur at this stage. By our late 20s, almost a third of us have well-developed lesions. (**4**) Finally, chronic inflammation and necrosis can lead to plaque rupture. Subsequent thrombosis may obstruct blood flow, often resulting in adverse clinical outcomes, including heart attack and stroke. Such clinical complications associated with complex atherosclerotic lesions can occur by middle age [2,3,5,6].

Cardiovascular disease (CVD) is the leading cause of death worldwide, costing the National Health Service in England an estimated £7.4 billion per year [1]. CVD is predominantly associated with atherosclerosis, which can typically be classified into four main stages (Figure 1).

(1) Firstly, endothelial cell (EC) activation, commonly occurring in areas of aberrant blood flow, stimulates the accumulation and oxidation of low-density lipoprotein (LDL) within the vessel wall [2]. Oxidised LDL and adhesion molecules secreted from activated ECs attract monocytes from the blood into the subendothelial intima where they differentiate into macrophages, which ingest lipoproteins and subsequently become foam cells [2]. These deposits, referred to as fatty streaks, begin to develop in childhood [3].

(2) In humans, vascular smooth muscle cell (VSMC) accumulation in the intimal space occurs in utero in conserved locations susceptible to the development of atherosclerotic plaque, including branch sites and areas of turbulent blood flow [4]. In response to inflammatory mediators and cytokines, VSMCs migrate, proliferate, and secrete extracellular matrix (ECM) proteins [2]. ECs have also been reported to lose cell-cell interaction and undergo endothelial to mesenchymal transition (EndoMT), contributing to plaque growth [2]. By the age of puberty, more than 50% of children present fibrous atherosclerotic plaque, containing hyperplastic VSMCs [3].

(3) In progressing plaques, macrophages and VSMCs become necrotic, releasing lipids which accumulate within the centre of the lesion to form the necrotic core [2]. VSMCs migrate and proliferate to create the fibrous cap and secrete ECM components providing stability to the atherosclerotic lesion [2]. Lymphangiogenesis and neovascularisation may also occur at this stage [5,6]. By our late 20s, almost a third of us have well-developed lesions with large lipid-filled necrotic cores and thick fibromuscular caps [3].

(4) In advanced plaque, the fibrous cap may rupture and trigger the blood to clot—this can lead to complications including heart attack and stroke by middle age [3,7]. Percutaneous coronary intervention and bypass grafts are sometimes required to restore blood flow; however, such interventions themselves may lead to further clinical complications [8,9]. In recent years, numerous studies have revealed that ECs, macrophages, and VSMCs undergo extensive phenotypic changes in diseased blood vessels, often in response to lipid and pro-inflammatory cytokine exposure [10,11,12,13,14,15,16,17,18,19,20,21,22]. These phenotypic changes can both promote and attenuate the progression of occlusive vascular diseases including atherosclerosis, interventional restenosis, and transplant vasculopathy [10,11,12,13,14,15,16,17,18,19,20,21,22].

Neuropilin-1 (NRP1) and its closely related family member NRP2, are transmembrane co-receptors, which are devoid of kinase activity [23,24,25]. Both NRP family members are expressed by a wide variety of cell types [23,24,25]. Although NRPs are commonly found at the cell surface, they have also been reported within the mitochondria [26,27] and nucleus [28]. NRPs were first implicated in neuronal guidance, vasculogenesis and angiogenesis in the embryo via mediating signalling pathways driven by class 3 Semaphorins (SEMA3s) and Vascular Endothelial Growth Factor (VEGF), respectively [25,29,30,31,32]. However, NRPs are now known to partner with a wide variety of transmembrane receptors and therefore modulate numerous signalling pathways [23,24], including those activated by Epidermal Growth Factor (EGF) [33], Fibroblast Growth Factor (FGF) [34], Hepatocyte Growth Factor (HGF) [35], Insulin-like Growth Factor (IGF) [36], Platelet-Derived Growth Factor (PDGF) [37,38] and Transforming Growth Factor beta (TGFβ) [39]. As a result, NRPs mediate multiple cellular processes, and dysregulation of their activity has been implicated with several pathological conditions [23,38,40,41,42]. NRPs are expressed by ECs [31], leukocytes [24] and VSMCs [38,40,43,44,45] within the vasculature, and are emerging as multifaceted regulators of signalling pathways associated with CVD [23]. 

**Figure 2:**
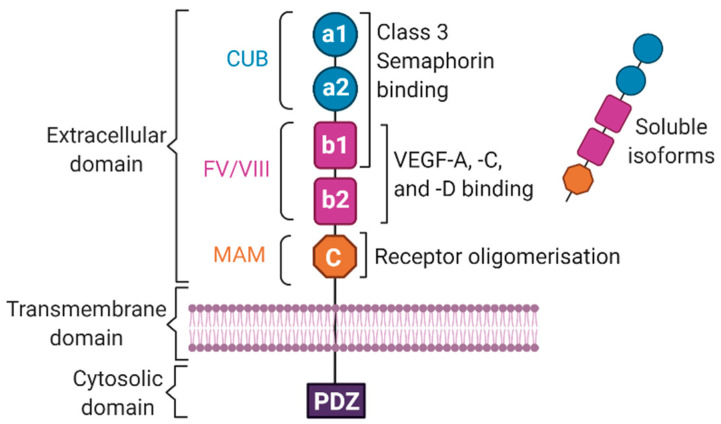
Basic Neuropilin (NRP) structure. NRPs have an extracellular domain comprised of two tandem CUB (complement C1r/C1s, Uegf, Bmp1) subunits (blue circles), two factor V/VIII coagulation factor homology domains (pink squares) and a MAM (meprin, A-5 protein, receptor protein-tyrosine phosphatase mu) subunit (orange octagon), a transmembrane domain, and a short cytoplasmic domain [24,46,47]. NRPs can also be found in soluble forms [27,47]. The a1/a2/b1 subunits interact with class 3 Semaphorins, the b1/b2 subunits interact with VEGF-A, -C, and -D, and the c subunit is thought to be involved in oligomerisation with other receptors [48,49,50]. The PDZ binding motif facilitates association with proteins containing a PDZ (PSD95, Dlg, ZO-1) domain, including GIPC (RGS-GAIP-interacting protein) [52,53,54].

Both NRP family members share the same basic structure consisting of an extracellular domain comprised of two CUB (complement C1r/C1s, Uegf, Bmp1) subunits (a1, a2), a factor V/VIII coagulation factor homology subunit (b1, b2) and a MAM (meprin, A-5 protein, receptor protein-tyrosine phosphatase mu) subunit (c), a transmembrane domain, and a short cytoplasmic domain (Figure 2) [24,46,47]. NRPs can also be found in soluble forms created by alternative splicing [47] or by extracellular domain shedding of the transmembrane proteins [27] (Figure 2). The a1/a2/b1 subunits have long been known to interact with class 3 Semaphorins to regulate neurogenesis while the b1/b2 domains bind to VEGF-A and VEGF-C to mediate angiogenesis [48]. Although less well characterised, VEGF-D has also been described to interact with NRP2 in the context of lymphangiogenesis [49]. The extracellular c domain was thought to be important for NRP oligomerisation with other cell surface receptors [48,50]; however, a recent study has challenged this function [51]. The intracellular PDZ (PSD95, Dlg, ZO-1) binding motif facilitates association with PDZ domain-containing proteins, including GIPC (RGS-GAIP-interacting protein), which can directly mediate signalling in ECs [52,53,54]. 

Despite being 44% identical at the amino acid level, NRP1 and NRP2 exhibit distinct expression patterns and ligand preferences in vivo [46,55]. For example, NRP1 predominantly binds to VEGF-A_165_ to mediate angiogenesis [56,57,58] in vascular ECs, whereas NRP2 interacts with VEGF-C to regulate lymphangiogenesis [46,59,60,61]. *NRP1* and *NRP2* knockout mice also display disparate phenotypes—while NRP1 knockout is embryonic lethal at E10.5 to E13.5 due to a spectrum of cardiovascular and neuronal defects, NRP2 knockout embryos remain viable but exhibit decreased numbers of lymphatic capillaries, as well as abnormal guidance and arrangement of cranial and spinal nerves [30,32,61,62]. NRP1 and NRP2 double knockout mice display even greater vascular defects and die in utero at E8.5 with large areas in the yolk sac totally void of blood vessels [63]. Furthermore, NRP1 and NRP2 are regulated by separate stimuli which elicit different cellular responses. For example, in smooth muscle cells (SMCs), while NRP1 appears to be upregulated by FGF and PDGF [38,64], which promote migration and proliferation [65,66], NRP2 is induced by Tumour Necrosis Factor alpha (TNFα) and Interleukin-1 beta (IL-1β), which elicit a pro-inflammatory response [66,67,68,69,70]. Together, these observations suggest that NRP1 and NRP2 have unique as well as overlapping functions. NRP1 is known to mediate vascular disease associated TGFβ and PDGF signalling as reviewed by Kofler and Simons [23]; however, the role of NRP2 is less well defined. Here, we discuss recent studies implicating NRP2 in the development of occlusive vascular diseases and consider how NRP2 could be targeted for therapeutic intervention.

## 2. Endothelial Cell Dysfunction

Disturbed blood flow results in EC activation, which refers to the attainment of a pro-inflammatory and pro-coagulant state associated with the initiation of atherogenesis (Figure 1). Although much is known regarding NRP1 function in EC migration, proliferation, and permeability [23,71,72], the role of NRP2 in EC dysfunction remains unclear. However, there is building evidence implicating EC-derived NRP2 in EndoMT, lymphangiogenesis, and neovascularisation. These processes are all associated with the development of occlusive vascular diseases.

### 2.1. Endothelial to Mesenchymal Transition

Following their activation, ECs further contribute to neointimal thickening in atherosclerosis by undergoing EndoMT. EC lineage tracing studies have shown that, in response to injury or inflammation, ECs lose the expression of EC markers such as VE-cadherin and CD31, and acquire myofibroblast markers, including α-smooth muscle actin, N-cadherin and calponin [17]. As well as the acquisition of a more contractile phenotype, EndoMT is accompanied by loss of cell-cell/cell-ECM contact, increased migratory activity, and synthesis of ECM components [17].

Both in vitro and in vivo studies have revealed that activation of the TGFβ signalling pathway is a major inducer of EndoMT [17,73]. In mouse pancreatic ECs, TGFβ-induced EndoMT upregulates miR27 levels, which post-transcriptionally suppresses NRP2 expression [74]. Since NRP2 deficiency promotes SMC contractility [43,70,75], the authors hypothesise that miR27-induced silencing of NRP2 activity is required for the transition to a more contractile phenotype [74]. However, the authors observed that miR27 also targets ELK1, a myogenic transcription factor which acts as a competitive inhibitor of serum response factor (SRF). Many contractile genes are regulated by SRF, a transcription factor which binds to CC(A/T-rich)_6_GG (CArG) *cis*-elements of all known CArG dependent contractile SMC genes, including *ACTA2*, *CNN1*, *TAGLN* and *MYH11* [18]. Therefore, miR27-induced silencing of NRP2 may not be causally linked to the transition to a more contractile phenotype in EndoMT but rather the modulation of contractile genes by SRF.

**Figure 3:**
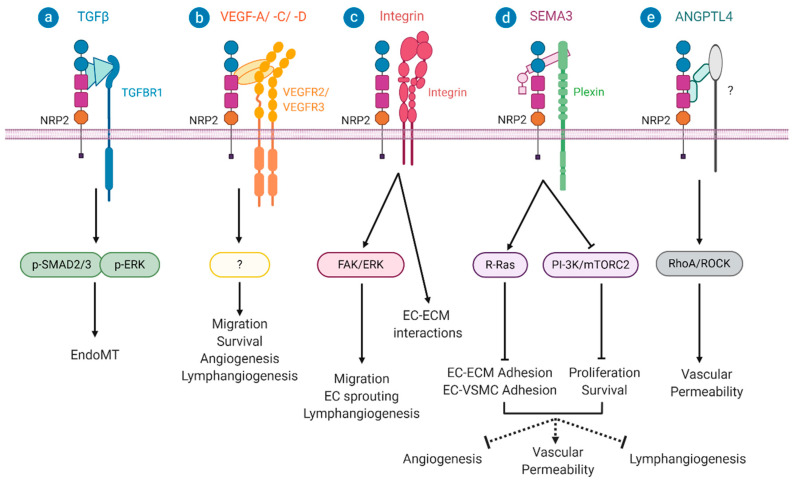
Endothelial cell NRP2-mediated signalling pathways. (**a**) NRP2 may positively regulate EndoMT by directly binding to TGFβ1 and complexing with TGFBR1 [39]. (**b**) NRP2 interacts with VEGFR2 and VEGFR3 to promote migration and survival induced by VEGF-A and VEGF-C [80]. NRP2 has also been found to interact with VEGF-D [49]. The sprouting response of lymphatic ECs to VEGF-C is mediated by NRP2 complexing with VEGFR-3 [81]. (**c**) VEGF/NRP2 signalling facilitates α6β1 integrin engagement with ECM components. Signalling through this integrin activates a FAK/ERK driven signalling resulting in EC migration, sprouting and lymphangiogenesis [84] (**d**) NRP2 complexes with Plexins to drive SEMA3 driven R-Ras signalling leading to loss of EC-ECM/VSMC adhesion which leads to increased vascular permeability [93,95]. NRP2/Sema3F/Plexin A1 inhibits PI-3K and mTORC signalling attenuating EC proliferation and survival [94]. The NRP2/SEMA3/Plexin complexes are implicated in downregulating angiogenesis and lymphangiogenesis, while promoting vascular permeability [91,92,93,94,95,96,97]. (**e**) NRP2 binding to ANGPTL4 drives the activation of the RhoA/ROCK signalling pathway to facilitate breakdown of EC-EC junctions and thus increase permeability [99].

Indeed, Grandclement et al. found that transfecting a colon cancer epithelial cell line (HT29 cells) with plasmids encoding *NRP2* induces an elongated fibroblast-like cell morphology reminiscent of mesenchymal cells [39]. The authors go on to show that NRP2 expression in HT29 cells activates TGFβ signalling, leading to constitutive phosphorylation of the Smad2/3 complex and inhibition of E-cadherin expression (an epithelial cell marker) and upregulation of vimentin expression (a protein specific to mesenchymal cells) [39]. The group used surface plasmon resonance to demonstrate that NRP2 binds to TGFβ1 and performed co-immunoprecipitation to show that NRP2 complexes with TGFBR1 in HT29 cells. Pharmacological inhibition of TGFBR1 restored epithelial cell markers and inhibited NRP2-induced mesenchymal gene induction in HT29 cells [39]. Therefore, NRP2 may positively regulate EndoMT by directly binding to TGFβ1 and complexing with TGFBR1 (Figure 3a). 

### 2.2. Lymphangiogenesis and Neovascularisation

Lymphatic vessels (LVs) are found in adventitial and intraplaque regions of human atherosclerotic carotid arteries [76]. There is evidence suggesting LVs are essential for the removal of cholesterol, leukocytes, and cytokines from atherosclerotic blood vessels [5]. As a result, LVs may help reduce plaque burden. In atherosclerotic lesion sites, local environmental cues also stimulate angiogenesis from pre-existing vasa vasorum, a network of small blood vessels that supply the walls of large arteries. Intraplaque neovascularisation facilitates the supply of nutrients and O_2_ to atherosclerotic lesions supporting plaque growth. However, dysregulated neo-capillary development may promote intraplaque haemorrhages increasing the chance of plaque rupture.

NRP2 is highly expressed in lymphatic ECs and in the stroma of many types of tumour, where it supports lymphangiogenesis and neovascularisation [77]. Furthermore, NRP2 deficiency suppresses lipopolysaccharide (LPS)-induced lymphangiogenesis [78] and VEGF-induced neovascularisation in the retina [79]. These observations suggest that NRP2 may play a role in lymphangiogenesis and neovascularisation associated with occlusive vascular diseases. In 2006, in vitro studies demonstrated that NRP2 interacts with VEGFR2 and VEGFR3 to promote human EC survival and migration induced by VEGF-A and VEGF-C [80]. Furthermore, in 2006, Kärpänen et al. found that NRP2 interacts with VEGF-C and VEGF-D and complexes with VEGFR3 in lymphatic ECs [49]. A couple of years later, Caunt et al. found that tumour associated lymphangiogenesis was reduced by blocking VEGF-C–NRP2 binding [59]. Using transgenic mice, Xu et al. demonstrated that the sprouting of lymphatic ECs in response to VEGF-C is mediated by NRP2 complexing with VEGFR3 [81] (Figure 3b). Further studies showed that TGFβ-mediated activation of TGFBR1 and TGFBR2 promotes dermal lymphatic EC sprouting by upregulating VEGFR3 and NRP2 expression [82].

NRP2 is also known to regulate lymphangiogenesis and angiogenesis via VEGF independent mechanisms. Indeed, recent studies have revealed that NRP2 interacts with integrins, a family of widely expressed cell adhesion receptors [83,84,85]. In particular, NRP2 has been shown to complex with integrin α5 [83] and α9 [84]—adhesion molecules which bind to proteins containing an arginine, glycine, and aspartate (RGD) amino acid sequence [85]. The RGD motif is common to numerous ECM proteins which display enhanced deposition during vascular remodelling responses, such as fibronectin, fibrinogen, and vitronectin [85]. Interestingly, EC integrin α5β1 interaction with fibronectin is implicated with activation of the CVD-associated NFkB signalling pathway [86,87,88] and atherosclerotic angiogenesis [89]. Cao et al. demonstrated that NRP2 expressed on cancer cells interact with α5 integrins on ECs to promote vascular adhesion and extravasation using renal and pancreatic cell cancer models [83]. Activation of the NFkB signalling pathway is responsible for the transcriptional induction of pro-inflammatory cytokines, chemokines, adhesion molecules, and genes which alter the composition of the ECM. The development of numerous cancers as well as vascular diseases are strongly associated with the expression of NFkB target genes. Therefore, it is worthwhile investigating whether the modulation of EC adhesion and migration on fibronectin by recycling integrin α5 via NRP2, as described in Alghamdi *et al*., is NFkB-dependent [90]. Furthermore, Ou et al. observed that, in colorectal carcinoma, expression of NRP2 in lymphatic ECs significantly increases their migration, sprouting, and tubulogenesis capacity via binding to integrin α9β1 and stimulating activation of the FAK/ERK signalling pathway [84] (Figure 3c). NRP2 may therefore modulate integrin-mediated vascular cell-ECM and cell-cell interactions to regulate signalling pathways, and fine-tune cell function to local environmental conditions.

NRP2 is also thought to play a role in blocking lymphangiogenesis and neovascularisation while promoting vascular permeability through interacting with Plexin receptors and binding to SEMA3s [91,92,93,94,95,96,97,98] (Figure 3d). There is evidence suggesting that SEMA3s may compete with VEGF family members for binding to NRP2 [91,97]. However, other studies have demonstrated that NRP2 complexes with Plexin receptors to promote SEMA3 driven R-Ras signalling, leading to the inactivation of β1-integrin and dissociation from the ECM [93,98]. As a result, SEMA3s are thought to inhibit angiogenesis via impairing EC adhesion after binding to NRP2. SEMA3G stimulation, a ligand known to bind to NRP2, of EC and SMC co-cultures leads to rapid EC detachment [95]. NRP2 interaction with Sema3F and Plexin A1 has also been shown to inhibit PI-3K and mTORC signalling attenuating EC proliferation and survival [94]. There is evidence suggesting that loss of endogenous Sema3F activity is responsible for enhanced vascular permeability, inflammation, and lymphedema observed in inflamed ears of NRP2 deficient mice compared to wild type controls [96]. 

Finally, NRP2 also binds to Angiopoietin-like 4 (ANGPTL4), a multifunctional protein involved in wound healing and modulation of vascular permeability. The resulting activation of the complex leads to the activation of the RhoA/ROCK signalling pathway, the dissociation of EC-EC junctions, and subsequent retinal vascular leakage [99] (Figure 3e).

With these observations in mind, it is tempting to speculate that NRP2 influences changes in EC behaviour associated with CVD. However, whether it is beneficial to target EC-derived NRP2 to treat occlusive vascular diseases is most likely context dependent. Therefore, more studies are required to further our understanding of EC-derived NRP2 in the context of CVD.

## 3. Monocyte Recruitment and Macrophage Activity

### 3.1. Monocyte Recruitment

Atherogenesis involves the recruitment of circulating monocytes to the vascular wall. Once adhered to the vascular wall, monocytes differentiate into macrophages, ingest lipoproteins, and subsequently become foam cells (Figure 1) [100]. This process helps clear atherogenic lipoprotein complexes from the blood vessel; however, excessive oxLDL uptake has adverse effects [100]. Lipid-rich foam cells secrete pro-inflammatory cytokines, elicit pro-apoptotic pathways, and inhibit clearance of dying cells leading to lesion growth and destabilisation of the necrotic core [100]. Interestingly, leukocytes, as well as ECs and VSMCs, express integrins, which are known to interact with NRPs [85,101]. As activated ECs [82] and VSMCs [38,68,69,70] upregulate NRP2 expression, NRP2 may mediate monocyte adhesion to the vascular wall by interacting with integrins expressed by monocytes. 

### 3.2. Macrophage Activity

Lipid-laden macrophages are a key feature of atherosclerotic lesions. Plaque resident macrophages originate from numerous sources including circulating bone marrow-derived monocytes, local proliferation of tissue-resident macrophages, and transdifferentiation of VSMCs [11,12]. Indeed, single-cell transcriptomic analysis revealed that macrophages within atherosclerotic plaque are extremely heterogenous—this observation may help explain how they perform multiple functions, which influence atherogenesis and lesion stability [11,12].

Interestingly, NRP2 is not initially detectable in monocytes but becomes highly expressed as they differentiate into macrophages [102,103]. NRP2 is highly and broadly expressed by different macrophage subsets, including alveolar, bronchial, peritoneal, and intravascular macrophages [24,102,104]. This observation suggests that NRP2 may play an important role in macrophage function.

**Figure 4:**
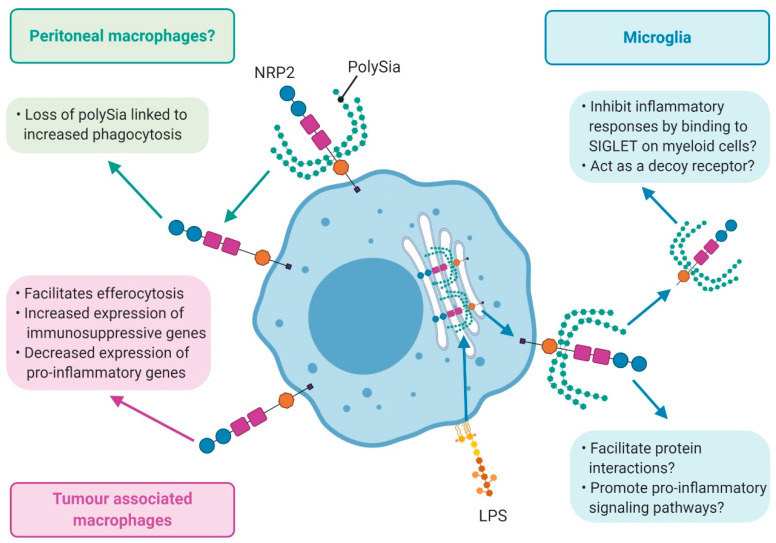
Role of NRP2 in macrophages. In peritoneal macrophages (green boxes), NRP2 is implicated in phagocytosis [105]. In tumour-associated macrophages (TAMs) (pink boxes), NRP2 facilitates efferocytosis whilst promoting the expression of pro-inflammatory genes [103]. In microglia (blue boxes), following LPS challenge, polysialylated NRP2 rapidly translocates to the cell surface before being shed from the cells [27]. Soluble polysialylated NRP2 may have an immunosuppressive role by binding to SIGLET to attenuate activation of pro-inflammatory signalling pathways [106,107]. However, polysialylated NRP2 at the cell surface may facilitate protein interactions to promote pro-inflammatory signalling.

Roy et al. observed that NRP2 expression in macrophages correlates with their ability to perform efferocytosis, a process by which apoptotic cells are engulfed and cleared by macrophages [103]. NRP2 inhibition using siRNA attenuated phagosome maturation without affecting uptake of phagocytic cargo in macrophages derived from human peripheral blood mononuclear cells treated with colony-stimulating factor (CSF) [103]. Furthermore, NRP2 knockout specifically in bone marrow-derived macrophages attenuated the clearance of apoptotic cells in vitro [103]. These observations were validated *in vivo*; in the same study, they demonstrated that the ablation of NRP2 in tumour-associated macrophages (TAMs) impaired the clearance of apoptotic tumour cells, resulting in elevated levels of necrosis within the tumour core [103]. Interestingly, the group also observed downregulation of immunosuppressive genes (IL4, IL10, MMP11, MMP13) and upregulation of immunostimulatory genes (IL12a, IFNb, Gr-K, Gr-F) following NRP2 deletion in TAMs [103] (Figure 4). Therefore, it is tempting to speculate that NRP2 enables macrophages to efferocytose without causing an immune response against apoptotic tumour cell components. It would be worthwhile investigating whether NRP2+ macrophages, in a similar manner, help clear lipids and dying cells from atherosclerotic plaque without eliciting inflammation. If so, NRP2+ macrophages could promote atherosclerotic plaque regression and stability.

Intriguingly, NRP2 is one of only six proteins which can be post-translationally modified by the addition of polysialic acid (polysialylation) and removal of polySia enhances phagocytosis of *Klebsiella pneumoniae* by peritoneal macrophages [105]. Furthermore, peritoneal macrophages isolated from inflamed sites express NRP2 which has not been modified by polysialylation but start to re-express polySia on NRP2 after 24 h in culture when removed from the inflammatory environment [105]. This observation suggests NRP2 facilitates phagocytosis in peritoneal macrophages although a direct causal link has not yet been demonstrated (Figure 4). Therefore, in the context of atherosclerosis, loss of polySia on macrophage-derived NRP2 may facilitate phagocytosis of lipids and apoptotic cells.

Polysialylated NRP2 is shed by microglia in response to LPS-induced conversion to a pro-inflammatory phenotypic state (Figure 4). LPS stimulation of cultured microglial cells induces translocation of polysialylated NRP2 from Golgi-confined intracellular pools to the cell surface before polysialylated NRP2 is cleaved and shed into the supernatant [27] (Figure 4). Because polysialylated NRP2 is presented at the cell surface during early LPS challenge it may mediate interactions with signalling proteins which promote a pro-inflammatory phenotype; however, this is yet to be investigated (Figure 4). Conversely, polysialic acid is known to inhibit inflammatory responses by binding to sialic acid-binding immoglobulin-like lectin 11 receptor (SIGLET1) on human myeloid cells [106]. Polysialic binding to SIGLET1 initiates recruitment of cytoplasmic phosphatases to block signal transduction through dephosphorylation of signalling molecules [106]. Therefore, polysialated soluble NRP2 may act as an anti-inflammatory mediator [107] (Figure 4). Soluble forms of NRP2 are also known to act as decoy receptors. For example, a soluble form of NRP2 has been shown to scavenge VEGF-C and inhibit pathological NRP2/VEGF-C signalling in a prostate cancer cell line [108]. Further studies are required to elucidate the role of macrophage-derived NRP2, including post-translationally-modified, full length, and soluble isoforms, in phagocytosis and inflammation associated with CVD.

## 4. VSMC Phenotypic Switching

In recent years, numerous studies have demonstrated that VSMCs play a substantial role in atherosclerosis [18]. In the healthy blood vessel, VSMCs predominantly exhibit a quiescent “contractile” phenotype but can switch to a more active “synthetic” state which exhibits pronounced migratory and proliferative activity [18]. Upon insult, VSMCs downregulate the expression of contractile VSMC markers and upregulate genes required for vascular remodelling including cytokines, adhesion molecules, and proteins which alter the composition of the ECM [18]. VSMCs can also express markers typically associated with other cell types including macrophages, myofibroblasts, MSCs, and osteochondrocytes [18]. Strikingly, genetic lineage tracing studies have shown that up to 90% of plaque-resident cells are VSMC-derived [15,16].

### 4.1. NRPs are Expressed by Cardiovascular Precursor Cells

Ding et al. demonstrated that in murine embryonic stem cells (mESCs), upon BMP4 stimulation, NRPs are often co-expressed with Brachyury, a critical mesoderm inducing factor. NRP2 expression is induced in mesodermal precursor cell subpopulations capable of differentiating into cardiomyocytes, endothelium, and VSMCs. By inhibiting NRP2 expression in mESCs, the group demonstrated that NRP2 is functionally required for differentiation towards endothelium and VSMCs but not toward cardiomyocytes [109]. A study assessing the onset of NRP2 expression in early chick embryos supports this in vitro finding. In the chick embryo, NRP2 expression is found in cardiovascular precursor cells derived from the blood islands, primitive streak, and lateral plate mesoderm [110]. Additionally, during avian blood vessel development, NRP1 is predominantly expressed in the arteries whereas NRP2 is expressed in the veins [110]. Furthermore, by crossing mice containing Cre recombinase driven by the NRP2 locus with a ROSA26-LacZ reporter line, researchers have demonstrated that at E12.5, NRP2 is expressed mainly in the venous vasculature, while NRP1 expression is more arterial [111]. Interestingly, in vitro analyses of rabbit VSMCs demonstrated that venous VSMCs display a more de-differentiated phenotypic state and exhibit increased proliferative and synthetic activity in comparison to arterial VSMCs [112].

**Figure 5:**
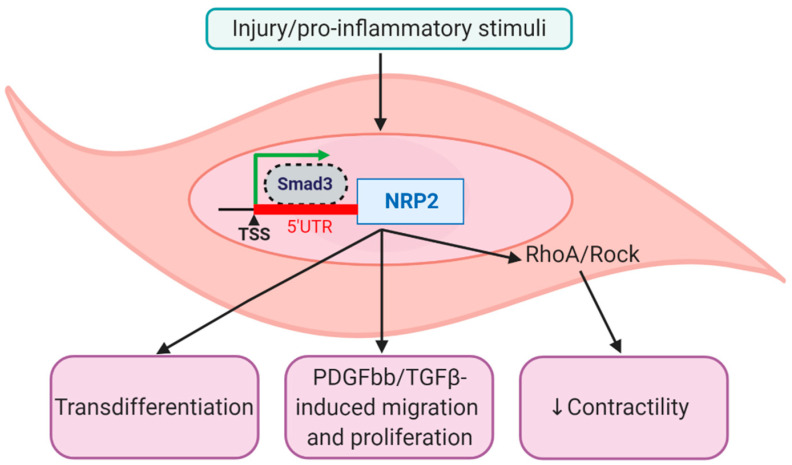
Roles of NRP2 in SMC phenotypic switching. NRP2 expression is upregulated in SMCs in response to injury [38,114] and pro-inflammatory stimuli [69,70] via Smad3 transcription factor binding to the NRP2 promoter [41]. NRP2 expression is linked to VSMC transdifferentiation [41,42]. Evidence suggests NRP2 positively regulates PDGFbb-induced VSMC migration and proliferation [38]. NRP2 is linked to loss of the contractile phenotype via promoting activation of the RhoA/ROCK signalling pathway [43,70,75]. TSS; transcriptional start site, UTR; untranslated region.

As cardiovascular precursor cells differentiate toward VSMCs, NRP2 expression is lost [109]. However, NRP2 expression can be re-induced in VSMCs in response to insult [38,67]. With these observations in mind, it is tempting to speculate that NRP2 marks a more dedifferentiated VSMC—a hallmark of many occlusive vascular diseases. In support of this theory, NRP2 promotes stem-like traits in breast cancer cells via VEGF-driven signalling [113]. Importantly, although the analysis is not yet peer-reviewed, new single-cell transcriptomic data of human atherosclerotic plaque also implicates NRP2 in VSMC transdifferentiation within the core [42] (Figure 5). It would, therefore, be of great scientific and therapeutic interest to investigate whether NRP2 expression marks a more plastic VSMC state.

### 4.2. Nrp2 is Re-Expressed in Mature VSMCs in Response to Injury/Inflammation

VSMCs do not appear to express NRP2 under quiescent conditions [43] but increased levels of NRP2 have been observed in SMCs in response to injury [38,41,114] and inflammatory stimuli [66,67,68,69,70] (Figure 5). Elevated NRP2 expression has been detected within VSMCs following balloon angioplasty in a rat model of vascular injury [38]. This increase was particularly noticeable at time points known to correlate with increased levels of pro-inflammatory cytokines within the vessel wall [115,116]. Downregulation of NRP2 using shRNA adenoviral vectors resulted in a significant reduction of neointimal hyperplasia following balloon angioplasty, supporting a role for NRP2 in disease-associated vascular remodelling [38]. NRP2 inhibition was also shown to reduce PDGFbb-mediated migration and proliferation of rat VSMCs in vitro as well as injury-induced rat VSMC proliferation in vivo [38] (Figure 5). However, NRP2 levels were not influenced by PDGFbb stimulation. Xie et al. observed that NRP2 expression in VSMCs in response to injury is induced by p-SMAD3 binding to the 5′ untranslated region (UTR) of NRP2, between +51 to +78bp from the transcriptional start site (TSS) [41]. Importantly, using siRNA to inhibit NRP2 activity, the group demonstrate that, in primary human aortic SMCs, NRP2 positively regulates TGFβ-induced VSMC proliferation and migration as well as cholesterol-induced conversion to a macrophage-like (CD68+) phenotype [41] (Figure 5). 

Numerous studies have demonstrated that, in response to inflammation, VSMCs downregulate the expression of contractile genes—a process associated with plaque instability [18]. In healthy adult mice, NRP2 is expressed by SMCs in the bladder and gut but not in the vasculature, heart, or skeletal muscle [43]. Interestingly, SMC-specific knockout of NRP2 promotes the contractility of SMCs in the bladder and gut via mediating activation of the RhoA/ROCK signalling pathway [43,70,75] (Figure 5). This finding suggests that injury/inflammation-induced NRP2 upregulation by VSMCs may be linked to loss of the contractile VSMC state. However, no studies to date have investigated if disease-associated loss of VSMC contractility is causally linked to NRP2 expression. Additionally, while NRP2 expression is upregulated by pro-inflammatory stimuli known to activate the NFkB signalling pathway [68,69,70], contractile VSMC genes are downregulated [117,118,119,120], suggesting NRP2 may not directly cause loss of VSMC contractility. Activation of the NFkB signalling pathway in VSMCs eventuates in NFkB (p65 subunit) binding to the MYOCD promoter, decreasing the expression of MYOCD and MYOCD-dependent contractile genes (e.g., ACTA2, TAGLN, and MYH11) [117,118,119,120]. 

## 5. NRP2 is an Attractive Therapeutic Target

NRP2 is upregulated by vascular cell types in response to injury/inflammation and is involved in numerous processes associated with the development of occlusive vascular diseases. Currently, many molecules linked to CVD, including various transcription factors, are poor drug targets. Because cell surface receptors are more easily targeted, NRP2 could be a novel therapeutic candidate or biomarker to treat or detect diseased blood vessels. 

A variety of methods to inhibit VEGF-C/NRP2-driven signalling have been explored [59,108,121]. VEGF-C/NRP2 driven signalling promotes EC migration and survival as well as angiogenesis and lymphangiogenesis (Figure 3)—processes associated with vascular neointimal hyperplasia and atherosclerosis. Therefore, inhibiting VEGF-C/NRP2 driven signalling could potentially be used therapeutically in the treatment of occlusive vascular diseases. Monoclonal antibodies targeting the VEGF-C binding domain of NRP2 have been developed and were able to decrease the number of tumour-associated lymphatic vessels and metastasis in animal xenograft experiments [59]. Furthermore, a soluble splice variant of NRP2, s9NRP2, has been shown to scavenge VEGF-C away from the functional complex and inhibit the formation of prostatospheres by a human prostate carcinoma cell line [108], suggesting that s9NRP2 could potentially be used to inhibit pathological VEGF-C/NRP2 signalling in prostate cancer [108]. In 2020, Said et al. have reported the discovery of a novel benzamidine-based inhibitor that functions via competitive inhibition of VEGF-C binding to NRP2 [121]. To date, no other pharmacological inhibitors have been identified which target NRP2.

However, atherogenesis is complex and whether VEGF-C/NRP2-driven cellular responses prevent or promote adverse clinical outcomes is most likely context-dependent. For example, local inhibition of VEGF-C/NRP2 driven signalling may block a deleterious EC response to injury by attenuating EC migration and survival and may, therefore, be a useful strategy to prevent re-occlusion of blood vessels after surgical intervention. However, as NRP2 promotes lymphangiogenesis, which is important for the removal of cholesterol, leukocytes, and cytokines from plaque, inhibiting NRP2 in advanced atherosclerotic lesions may promote plaque growth and instability.

Additionally, because NRP2 promotes PDGFbb and TGFβ-induced VSMC migration, proliferation, and loss of SMC contractility, it may be beneficial to inhibit NRP2 as a therapeutic strategy to treat occlusive vascular diseases. NRP2 could be inhibited by developing neutralising antibodies. Promisingly, directly inhibiting PCSK9 (a protein which promotes levels of circulating LDLs) and IL-1β (a pro-inflammatory cytokine) using monoclonal antibodies reduced the rate of cardiovascular events, including heart attack and stroke, compared to placebo in patients [122,123,124]. However, NRP2 inhibition may also have a deleterious effect as NRP2 could potentially play an important role in the phagocytosis of lipids by macrophages, helping clear atherogenic lipid complexes from the vascular wall. Once again, it is important to emphasise that NRP2 inhibition may promote or prevent clinical complications associated with CVD depending on the context. Consequently, a better understanding of NRP2 function in CVD is of potential therapeutic interest.

As NRP2 is upregulated in the vasculature in response to injury and inflammation, it could be used to help diagnose diseased arteries. For instance, Chen et al. developed an iodine-131 labelled NRP2 monoclonal antibody to image A549 xenograft tumours in mice by single-photon emission computed tomography (SPECT) [125]. As SPECT is used clinically to visualise atherosclerotic plaques [126], perhaps, a similar approach could be used to visualise obstructed blood vessels in patients.

## 6. Conclusions and Future Perspectives

To summarise, NRP2 is expressed by numerous cell types within the vasculature and plays multifunctional roles in processes associated with the development of occlusive vascular diseases. Recent studies implicate NRP2 in EndoMT, lymphangiogenesis, angiogenesis, monocyte recruitment, the ability of macrophages to phagocytose, and VSMC plasticity. Consequently, NRP2 is likely to have complex roles within atherosclerotic blood vessels and may function to both prevent and promote plaque stability depending on the context. NRP2 is already targeted for cancer therapy [127]. Since cancer and occlusive vascular diseases share many commonalities, including pronounced cell plasticity, migration, proliferation, and inflammation [6], NRP2, an accessible cell surface receptor, may be a novel candidate for therapeutic intervention. However, the role of NRP2 in CVD remains enigmatic—a detailed mechanistic characterisation of NRP2 function in CVD is required to develop efficient strategies to limit cardiovascular risk.
